# P09 Activity of newer antibiotics against NDM- and OXA-48-producing organisms

**DOI:** 10.1093/jacamr/dlac053.009

**Published:** 2022-05-31

**Authors:** Hodman Mohamed, Emmanuel Q Wey, Zahra Sadouki, Kerry Roulston, Alan Williams, Indran Balakrishnan

**Affiliations:** 1 University College London, London, UK; 2 Royal Free London NHS Trust and University College London, London, UK; 3 Royal Free London NHS Trust, London, UK; 4 Health Services Laboratories, London, UK

## Abstract

**Background:**

Several antibiotics active against carbapenemase-producing organisms have recently been licensed for clinical use. These organisms usually have multiple integrative and conjugative elements with different resistance genes. The positioning of these agents for empirical use in sepsis caused by different carbapenemase-producing organisms is important in optimizing the likelihood of appropriate treatment before susceptibility testing is complete. Here we present the activities of meropenem-vaborbactam, imipenem-relebactam, eravacycline and tigecycline against NDM- and OXA-48-producing clinical isolates in a London teaching hospital.

**Methods:**

Sixty-two previously characterized carbapenemase-producing Gram-negative clinical isolates were studied. These comprised 31 NDM and 31 OXA-48 producers. OXA-48 producers comprised *Klebsiella pneumoniae* (17), *Escherichia coli* (10), *Enterobacter aerogenes* (3) and *Serratia marcescens* (1). NDM producers comprised *K. pneumoniae* (11), *E. coli* (10), *Acinetobacter baumannii* (3), *Klebsiella oxytoca* (1), *Enterobacter asburiae* (1), *Enterobacter cloacae* (1), *Pseudomonas aeruginosa* (1), *Citrobacter koseri* (1), *Citrobacter freundii* (1) and *Leclercia adecarboxylata* (1). MICs of meropenem/vaborbactam, imipenem/relebactam, eravacycline and tigecycline were determined using Etests. Interpretation was done using EUCAST breakpoints.

**Results:**

*OXA-48 producers:* Eight of 17 *K. pneumoniae*, 8/10 *E. coli* and 2/3 *E. aerogenes* isolates were meropenem/vaborbactam susceptible. Of these, 2 *K. pneumoniae*, 5 *E. coli* and 1 *E. aerogenes* isolates were imipenem/relebactam susceptible. All imipenem/relebactam/susceptible isolates were also meropenem/vaborbactam susceptible. The *S. marcescens* isolate (1/1) was resistant to both antibiotics. The only isolates demonstrating susceptibility to the glycylcyclines were *E. coli*. Five of 10 were susceptible to both glycylcyclines. One of 10 was susceptible to one glycylcycline but resistant to the other. *NDM producers:* Five of 11 *K. pneumoniae*, 6/10 *E. coli* and 1/1 *K. oxytoca, E. asburiae* and *C. koseri* isolates were susceptible to meropenem/vaborbactam. Of these, two *K. pneumoniae* and the *K. oxytoca* (1/1) and *E. asburiae* (1/1) isolates were imipenem/relebactam susceptible. The *L. adecarboxylata* isolate (1/1) was meropenem/vaborbactam resistant but imipenem/relebactam susceptible. All *P. aeruginosa*, *C. freundii*, *A. baumannii* and *E. cloacae* isolates were resistant to both meropenem/vaborbactam and imipenem/relebactam. Seven of 10 *E. coli* isolates were tigecycline susceptible of which 6 were also eravacycline susceptible. One of 11 *K. pneumoniae*, 1/1 *K. oxytoca* and 1/1 *L. adecarboxylata* isolates were susceptible to both glycylcyclines. All other isolates were resistant to both glycylcyclines.

**Conclusions:**

In both OXA-48 and NDM producers, the activity of the four antibiotics tested was better against *E. coli* than *K. pneumoniae*. Meropenem/vaborbactam provided better cover than imipenem/relebactam. Eravacycline and tigecycline showed similar activity to each other, but their activity was inferior compared with the β-lactam combinations, particularly against *K. pneumoniae*.

